# Reduction in femoral neck and total hip bone mineral density following hospitalisation for diabetes-related foot ulceration

**DOI:** 10.1038/s41598-021-02233-y

**Published:** 2021-11-23

**Authors:** Marcel M. Nejatian, Salar Sobhi, Blake N. Sanchez, Kathryn Linn, Laurens Manning, Shuen-Chyn Soh, Jonathan Hiew, J. Carsten Ritter, Bu B. Yeap, Emma J. Hamilton

**Affiliations:** 1 Medical School, The University of Western Australia, Perth, Australia; 2 Department of Nuclear Medicine, Fiona Stanley Hospital, Perth, Australia; 3 Infectious Diseases Department, Fiona Stanley Hospital, Perth, Australia; 4 Multidisciplinary Diabetes Foot Unit, Fiona Stanley Hospital, Perth, Australia; 5 Department of Podiatry, Fiona Stanley Hospital, Perth, Australia; 6 Department of Vascular Surgery, Fiona Stanley Hospital, Perth, Australia; 7 Faculty of Health Sciences, Curtin University, Perth, Australia; 8 Department of Endocrinology and Diabetes, Fiona Stanley Hospital, CD-09, 11 Robin Warren Drive, Murdoch, Perth, 6150 Australia

**Keywords:** Endocrinology, Endocrine system and metabolic diseases, Diabetes, Diabetes complications

## Abstract

Management of diabetes-related foot ulceration (DFU) includes pressure offloading resulting in a period of reduced activity. The metabolic effects of this are unknown. This study aims to investigate changes in bone mineral density (BMD) and body composition 12 weeks after hospitalisation for DFU. A longitudinal, prospective, observational study of 22 people hospitalised for DFU was conducted. Total body, lumbar spine, hip and forearm BMD, and total lean and fat mass were measured by dual-energy X-ray absorptiometry (DXA) during and 12 weeks after hospitalisation for DFU. Significant losses in total hip BMD of the ipsilateral limb (− 1.7%, *p* < 0.001), total hip BMD of the contralateral limb (− 1.4%, *p* = 0.005), femoral neck BMD of the ipsilateral limb (− 2.8%, *p* < 0.001) and femoral neck BMD of the contralateral limb (− 2.2%, *p* = 0.008) were observed after 12 weeks. Lumbar spine and forearm BMD were unchanged. HbA1c improved from 75 mmol/mol (9.2%) to 64 mmol/mol (8.0%) (*p* = 0.002). No significant changes to lean and fat mass were demonstrated. Total hip and femoral neck BMD decreased bilaterally 12 weeks after hospitalisation for DFU. Future research is required to confirm the persistence and clinical implications of these losses.

## Introduction

Diabetes-related foot ulceration (DFU) is extremely burdensome, associated with substantial morbidity, high costs and premature death^1^. Over half of all DFUs will become infected, and 20% of these will require a lower extremity amputation^2^. Pressure offloading is one of the cornerstones of DFU management^3,4^. In addition to the use of offloading footwear devices, people with DFU are typically advised to limit physical activity to promote ulcer healing, particularly for plantar wounds^3,4^. It is possible that these DFU management strategies may negatively impact bone mineral density (BMD) and body composition, however existing data is limited.

Both type 1 and type 2 diabetes are associated with an increased risk of fractures, particularly at the hip^5–7^. Type 1 diabetes is associated with reduced BMD and a marked increase in hip fracture risk^5,7^. In contrast BMD is usually normal or increased in people with type 2 diabetes with a modestly increased hip fracture risk (attenuated by the competing risk of death)^5,6^. The bone fragility observed in diabetes is likely multifactorial due to factors such as impaired bone quality, visual impairment, and risk of falls as well as effects on BMD^5,8^ and has been associated with increased mortality^9^. People with DFU could potentially be at increased risk of fracture as a result of diabetes complications and comorbidities, however there is currently no published data on bone health specifically in people with DFU.

People with type 2 diabetes tend to have lower muscle mass and higher fat mass than those without diabetes^10^. The relationship between obesity and risk of DFU is complex and incompletely understood. The prevalence of low muscle mass in people with type 2 diabetes with DFU has been reported to be more than double that in people with type 2 diabetes without DFU independent of age and diabetes duration^11^. It is possible that low muscle mass and increased fat mass may be a consequence of DFU and/or DFU treatment, however prospective data is lacking.

People with DFU have a 5 year mortality comparable with many common cancers^1^. It is possible that interventions that improve wound healing in the short term may be associated with complications such as reduced bone mineral density, fractures, sarcopenia and increased central adiposity which may increase morbidity and mortality in the longer term. To date, there have been no longitudinal studies investigating changes in BMD and body composition in people with DFU. Therefore, this study aimed to investigate changes in BMD, and lean and fat mass during and 12 weeks after hospitalisation for DFU. The identification of changes in these variables may help guide the implementation of measures to improve outcomes and reduce the burden associated with this complication of diabetes.

## Participants and methods

### Study design and setting

This prospective, observational study was conducted from November 2018 to February 2020. Participants with a DFU requiring hospitalisation were recruited from the inpatient Multidisciplinary Diabetes Foot Unit (MDFU) at Fiona Stanley Hospital, a tertiary teaching hospital in Perth, Western Australia. The MDFU is an interdisciplinary team comprising endocrinologists, vascular surgeons, infectious diseases physicians and podiatrists, managing diabetes-related foot complications across both inpatient and outpatient settings. Treatment was standardised according to international guidelines, including debridement or amputation procedures for ulcers which were unable to be managed conservatively with offloading and antibiotics^4^. Revascularisation was performed when indicated.

This study was conducted according to the guidelines of the Declaration of Helsinki and approved by the South Metropolitan Health Service Human Research Ethics Committee (No. RGS1289). Written informed consent was obtained from all participants in the study.

### Participants

Participants were eligible if they were English speaking, aged 18 years or older, and had capacity to provide informed consent. People were excluded from the study if they had a prior hospital admission for the same DFU, were already using offloading footwear devices and/or reducing activity for the same DFU or a DFU at another site, were pregnant, or had an Eastern Cooperative Oncology Group status of 4 or greater. Signed, informed consent was obtained from each participant.

### Baseline characteristics

Baseline characteristics were recorded including demographics, comorbidities, and past and current microvascular and macrovascular complications of diabetes. Area of residence was categorised using the Australian Rural, Remote, and Metropolitan Area classification. The duration of each participant’s admission and their respective treatments were also recorded.

### Study variables

Baseline values of each variable were measured within 1 week of admission. Follow-up values were obtained 12 weeks after admission.

Body weight was measured using a calibrated digital scale. BMD of the lumbar vertebrae (mean of L1–L4), ipsilateral (in relation to the DFU) and contralateral total hip and femoral neck and body composition were measured with dual-energy X-ray absorptiometry (DXA) using a Hologic Discovery A densitometer and APEX software version 4.0.2 (Hologic Inc., Marlborough, MA, USA). The scans were conducted by the same chief nuclear medicine technologist, and the machine was calibrated as per manufacturer’s instructions, including daily phantom scanning. In vivo BMD precision testing performed as per the International Society of Clinical Densitometry recommendations determined the BMD precision error (root mean square standard deviation) to be 0.01 g/cm^2^.

Grip strength was measured using a Jamar Plus+ hand dynamometer (Sammons Preston, Bolingbrook, IL, USA), calibrated within the last 12 months as per the manufacturer’s instructions. Standardised positioning and verbal encouragement as recommended by the American Society of Hand Therapists was used^12^. The maximal value of three attempts on each side were recorded, with 15 s rest between each attempt. The final grip strength reported was an average of the maximal grip strengths of the left and right sides.

Health-related quality of life was measured using the 36-Item Short Form Health Survey (SF-36)^13^. Ulcer area was measured at baseline and 12 weeks using the Silhouette Star wound measurement device (ARANZ Medical Limited, Christchurch, Canterbury, NZ).

### Statistical analysis

Results were analysed using R (V4.0.0; R Core Team, Vienna, Austria) and graphs were prepared using GraphPad Prism (V6.05; Graph Pad Software Inc, San Diego, CA, USA). A Shapiro–Wilk test was used for continuous variables to confirm a parametric distribution. Univariate comparisons of categorical data and paired continuous data were analysed using a Chi-squared test and paired *t* test, respectively. Pearson or Spearman correlation tests were used to explore associations between continuous variables for parametric and non-parametric data, respectively. Multivariate analyses were not performed. A *p *value < 0.05 was considered statistically significant.

## Results

### Characteristics of the study cohort

31 participants hospitalised for DFU management were recruited to the study. Nine participants did not complete their follow-up DXA scan resulting in data analysis from a total of 22 participants (18 men and 4 women). The majority (6 out of 9) of the participants reported difficulties with travel from a rural area as the main reason for incomplete follow-up. Demographic and clinical characteristics of the participants at baseline are shown (Table 1). The mean age was 61.7 years, the mean duration of diabetes was 16.1 years, and mean HbA1c was 75 mmol/mol (9.2%). All participants received inpatient antibiotic treatment and 16 participants (73%) underwent surgical intervention (either minor amputation and/or debridement). Two participants required revascularisation with an angioplasty. Three participants were placed in a knee-high removable offloading device, 15 in an ankle-high removable offloading device, and 4 participants continued using their normal or custom footwear. The majority of the participants (20 out of 22) were advised to partially weight-bear until follow-up (Table 1). No participants were on anti-resorptive or anabolic therapy for osteoporosis, hormone replacement therapy or oral glucocorticoid medication.

**Table 1:** Baseline demographic and clinical characteristics of 22 participants hospitalised with a diabetes-related foot ulcer (DFU). Data are presented as number (%) unless otherwise specified.

Parameter	Number (%)
Age (years)^1^	61.7 ± 1.9
**Gender**	
Male	18 (82)
Female	4 (18)
**Area of residence**	
Metro	14 (64)
Rural	8 (36)
Distance from hospital (km)^1^	59.6 ± 16.6
**Type of diabetes**	
Type 1	1 (5)
Type 2	21 (95)
Baseline HbA1c^1,2^ (mmol/mol, %)	75 ± 6, 9.2 ± 0.5
Length of diabetes (years)^1^	16.1 ± 2.0
**Co-morbidities**	
Previous DFU	15 (68)
Charcot foot	4 (18)
Diabetic retinopathy	13 (59)
Peripheral neuropathy	21 (95)
Chronic kidney disease	
Not diagnosed	17 (77)
Stage I	0
Stage II	2 (9)
Stage III	2 (9)
Stage IV	0
Stage V	1 (5)
Ischaemic heart disease	8 (36)
Previous myocardial infarction	3 (14)
Previous cerebrovascular accident	2 (9)
Hypertension	22 (100)
Dyslipidemia	19 (86)
**Smoking status**	
Current	4 (18)
Ex-smoker	6 (27)
Non-smoker	12 (55)
**Diabetes management on admission**	
Diet only	1 (5)
Oral agent(s) and/or non-insulin injectable only	8 (36)
Insulin with oral agent(s) and/or non-insulin injectable	11 (50)
Insulin only	2 (9)
**Diabetes management on discharge**	
Diet only	1 (5)
Oral agent(s) and/or non-insulin injectable only	7 (32)
Insulin with oral agent(s) and/or non-insulin injectable	12 (55)
Insulin only	2 (9)
**Affected limb of DFU**	
Left	15 (68)
Right	7 (32)
**Region of DFU on affected limb**	
Forefoot	15 (68)
Midfoot	5 (23)
Hindfoot	2 (9)
**Wound characteristic, Ischemia, and foot Infection (WIfI) grade**	
I	2 (9)
II	5 (23)
III	8 (36)
IV	7 (32)
**Acute DFU-related complications at baseline**	
Cellulitis	18 (82)
Osteomyelitis	9 (41)
Tenosynovitis	2 (9)
Sepsis	1 (5)
**Biochemistry on hospitalisation**	
C-reactive protein (mg/L)^1^	99.0 ± 23.9
Creatinine (µmol/L)^1,4^	95.5 ± 7.8
Urea (mmol/L)^1^	11.3 ± 3.3
Length of hospitalisation (days)^1^	7.5 ± 1.1
**Interventions performed during hospitalisation**	
Offloading device at discharge	
Normal/custom footwear	4 (18)
Ankle-high removable	15 (68)
Knee-high removable	3 (14)
Weightbearing instructions at discharge	
As normal	1 (5)
Partial	20 (91)
Non-weightbearing	1 (5)
Antibiotics	22 (100)
PICC line insertion	6 (27)
Debridement	13 (59)
Minor amputation	13 (59)
Revascularisation procedure	
Angioplasty	2 (9)
Healed DFU at 12-week follow-up^3^	6 (32)

^1^Data presented as mean ± SEM.

^2^Based on an analysis on *n* = 21.

^3^Based on an analysis on *n* = 19.

^4^A participant with stage V chronic kidney disease on dialysis had a creatinine of 786 µmol/L and was excluded.

### Changes in bone mineral density

**Figure 1:**
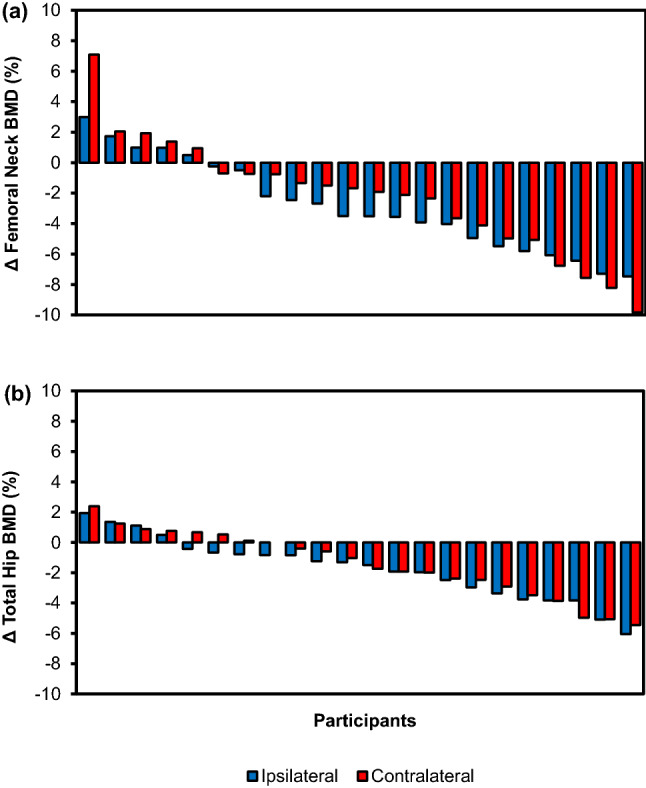
Percentage changes, in descending order, in ipsilateral and contralateral femoral neck BMD (**a**) and total hip BMD (**b**) of each participant 12 weeks following hospitalisation for a diabetes-related foot ulcer (*n* = 22).

A significant loss in total hip BMD of the ipsilateral limb (− 1.7%, *p* < 0.001), total hip BMD of the contralateral limb (− 1.4%, *p* = 0.005), femoral neck BMD of the ipsilateral limb (− 2.8%, *p* < 0.001) and femoral neck BMD of the contralateral limb (− 2.2%, *p* = 0.008) were observed at 12 weeks (Table 2). Most participants experienced BMD loss across both total hip and femoral neck sites, with the greatest decline observed at the ipsilateral femoral neck (Fig. 1). There were no significant differences in the loss of total hip or femoral neck BMD between ipsilateral and contralateral limbs (all *p* > 0.05). Surgical intervention (debridement and/or minor amputation) resulted in greater bone loss at the ipsilateral femoral neck compared to those who had non-surgical management (-3.7% vs -0.5%; *p* = 0.03). There was no difference in bone loss at the contralateral femoral neck or total hip in those patients requiring surgical intervention compared to those who had non-surgical management (all *p* ≥ 0.20). There was no significant change in BMD of the total body, lumbar spine, and distal third of the forearm after 12 weeks (all *p* > 0.05).

**Table 2:** Baseline and 12-week follow-up measurements of body mass index (BMI), total fat mass, total lean mass, grip strength, bone mineral density (BMD), wound area and HbA1c of 22 participants following hospitalisation for a diabetes-related foot ulcer (DFU). Data are presented as mean ± SEM unless otherwise specified.

Parameter	Baseline	12 Week follow-up	Change	% Change	*p* value*
BMI (kg/m^2^)	32.4 ± 1.2	33.1 ± 1.2	0.7 ± 0.5	2.1	0.20
Total body BMD (g/cm^2^)	1.261 ± 0.028	1.248 ± 0.024	− 0.013 ± 0.009	− 1.1	0.16
**Regional BMD (g/cm**^**2**^**)**					
Total hip					
Ipsilateral limb	1.023 ± 0.028	1.006 ± 0.029	− 0.017 ± 0.004	− 1.7	**< 0.001**
Contralateral limb	1.054 ± 0.029	1.51.039 ± 0.029	− 0.015 ± 0.005	− 1.4	**0.005**
Femoral neck					
Ipsilateral limb	0.872 ± 0.027	0.847 ± 0.027	− 0.025 ± 0.006	− 2.8	**< 0.001**
Contralateral limb	0.871 ± 0.027	0.852 ± 0.029	− 0.019 ± 0.006	− 2.2	**0.008**
Lumbar spine	1.214 ± 0.036	1.203 ± 0.038	− 0.012 ± 0.010	− 1.0	0.25
Distal third of forearm	0.765 ± 0.018	0.755 ± 0.018*	− 0.007 ± 0.04	− 1.2	0.08
**Bone disease category (number, %)**					
Normal	14 (64)	12 (55)	− 2	− 14	0.76
Osteopenia	8 (36)	9 (41)	1	13	
Osteoporosis	0 (0)	1 (5)	1	N/A	
Total fat mass (kg)	31.7 ± 1.8	32.1 ± 1.9	0.4 ± 0.55	1.2	0.50
Total lean mass (kg)	63.0 ± 2.0	64.2 ± 2.1	1.2 ± 0.92	1.8	0.22
Average grip strength (kg)	30.6 ± 2.0	29.2 ± 1.7*	− 0.14 ± 0.7	− 4.7	0.3
Wound area (cm^2^)	14.8 ± 6.1	7.6 ± 3.5	− 7.2 ± 3.8	− 49	0.08^1^
HbA1c (mmol/mol, %)	75 ± 6 (9.2 ± 0.5)	64 ± 3 (8.0 ± 0.3)	− 11 ± 4 (− 1.2 ± 0.8)	− 14	**0.002**^2^

Significant values are in bold.

*Paired *t* test.

^1^Based on a paired *t* test analysis on *n* = 19.

^2^Based on a paired *t* test analysis on *n* = 21.

There was no statistical association between patient demographics, clinical characteristics, previous history of DFUs, duration of diabetes, ulcer size, offloading device used, weight-bearing status, baseline BMD, baseline and changes in BMI, total fat and lean mass, and grip strength with observed changes in BMD (all *p* > 0.05; data not shown). Therefore, multivariate analyses of changes in BMD were not performed.

### Changes in body composition and grip strength

**Figure 2:**
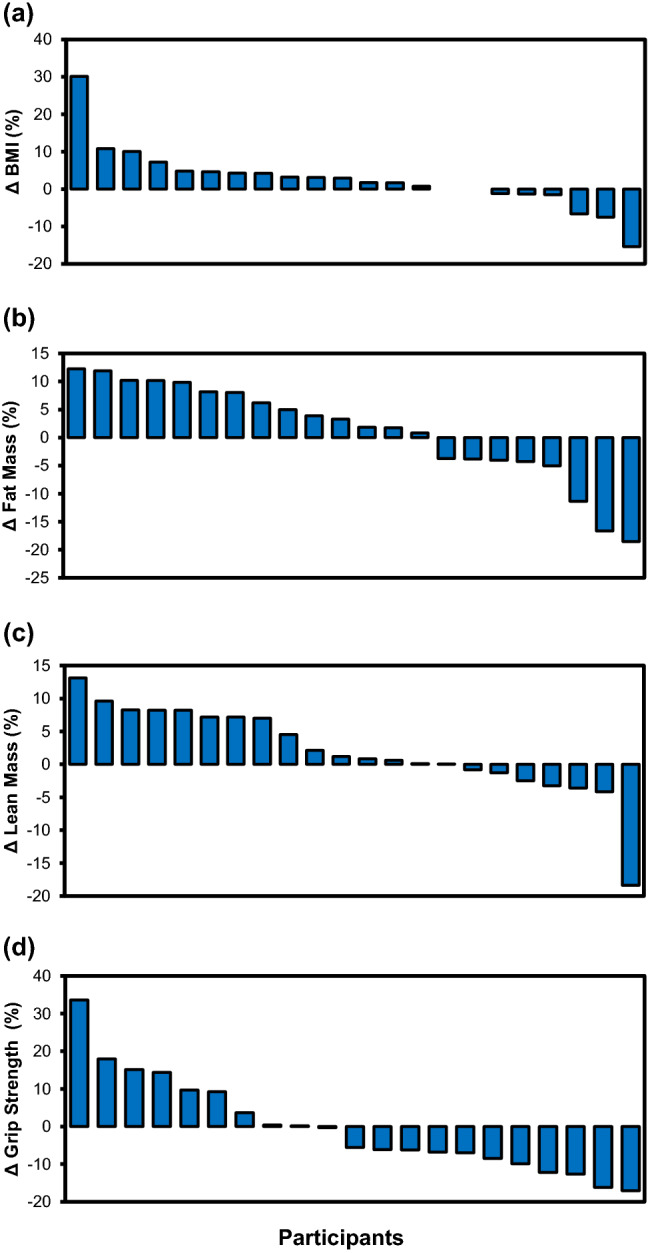
Percentage changes, in descending order, of BMI (**a**), fat mass (**b**), lean mass (**c**), and grip strength (**d**) of each participant 12 weeks following hospitalisation for a diabetes-related foot ulcer (*n* = 22).

There were no significant changes in body mass index (BMI), total fat mass, total lean mass, and grip strength of participants 12 weeks after enrolment (all *p* > 0.05; Table 2). Individually, most of the participants had an increase in their BMI, fat and lean mass, and a decrease in their grip strength (Fig. 2).

### Changes in quality of life and blood markers

There were no significant changes in the SF-36 domain scores at 12 weeks (all *p* > 0.05; Table 3). Participants had an improvement in their glycaemic control, with HbA1c decreasing by 11 mmol/mol (*p* = 0.002; Table 2).

**Table 3:** Average baseline and 12-week follow-up measurements of quality of life using the 36-Item Short Form Health Survey (SF-36) of 22 participants following hospitalisation for a diabetes-related foot ulcer. Data are presented as mean ± SEM.

Parameter	Baseline	12 Week follow-up	% Change	*p* value*
**SF-36**				
Physical functioning	55.2 ± 5.8	44.1 ± 5.3	− 20	0.10
Role limitation due to physical health	40.5 ± 9.2	26.2 ± 8.2	− 35	0.23
Role limitation due to emotional problems	61.9 ± 9.8	68.3 ± 9.0	10	0.56
Energy/fatigue	48.1 ± 4.7	54.3 ± 4.0	12	0.14
Emotional well-being	68.6 ± 4.6	76.2 ± 4.4	11	0.07
Social functioning	58.3 ± 7.1	65.5 ± 4.9	12	0.38
Pain	64.2 ± 6.6	64.8 ± 5.5	0.9	0.93
General health	43.1 ± 4.0	47.9 ± 5.0	11	0.20
Health change	42.9 ± 3.5	51.2 ± 5.6	19	0.29

*Paired *t* test analysis.

## Discussion

This is the first study to investigate longitudinal changes in BMD and body composition in people hospitalised for DFU and found significant losses in total hip and femoral neck BMD in both the ipsilateral and contralateral limbs over a 12-week period. There were no changes in total lean and fat mass, muscle strength, physical activity, or quality of life. The accelerated BMD losses we describe here are significant and may have important clinical implications for people with DFU.

There are few studies with which to directly compare our findings. Disuse osteoporosis is a well-documented phenomenon, which arises when skeletal loading is decreased or absent and results in inhibition of osteoblast mediated bone formation and excessive osteoclast mediated bone resorption^14^. This is typically observed in conditions in which profound, prolonged immobilisation and bed rest has occurred, such as critical illness, stroke and spinal cord injury^14^. Focal disuse osteoporosis has also been described, particularly in the setting of lower limb injuries requiring surgery and/or immobilisation with casting and a period of non-weight bearing and is often bilateral^14^. We also describe bilateral BMD losses, in which total hip and femoral neck BMD decreased by 1.7% and 2.8% on the ipsilateral side and by 1.4% and 2.2% on the contralateral side. Despite the trend towards greater losses on the ipsilateral side, the differences between the sides were not significant. Although greater ipsilateral femoral neck bone loss was observed in participants who underwent surgical intervention, the relevance of this result is unclear, given both surgically and non-surgically managed DFUs had similar bone losses at the contralateral femoral neck, and ipsilateral and contralateral total hip. While it is possible that the post-operative period and potentially greater reductions in mobility in patients with surgically managed DFUs may contribute to greater losses in BMD, this conclusion is beyond the scope and power of this study. Pakarinen et al. reported that after 6 months of immobilisation (11 weeks in a total contact cast followed by 12 weeks in an orthosis, combined with non-weight bearing through the affected limb with crutches or wheelchair) in acute Charcot neuroarthropathy (CNA), BMD of the ipsilateral femoral neck decreased by 3.2% and contralateral total hip total hip decreased by 1.2%^15^. Bilateral BMD losses also occurred over a 3 month period in adults managed with 6 weeks of plaster cast immobilisation and non-weight bearing following repair of a ruptured Achilles tendon^16^. These adults had a 3% and 1.8% loss of femoral neck BMD on the ipsilateral and contralateral sides, respectively, within the first 3 months post-repair, with no recovery at 1 year^16^. Similar bilateral losses in both total hip and femoral neck BMD have also been observed after 3 months of immobilisation following fractures of the lower leg, with limited recovery after 5 years^17^. None of these studies were conducted in the setting of acute DFU. In this study, participants with DFU utilised removable knee- and ankle-high offloading devices, were hospitalised for a mean duration of only 7.5 days and whilst physical activity and mobility were reduced, most participants were still able to stand upright and walk short distances on both feet with limited weight bearing permitted. This lesser degree of immobilisation and offloading was still associated with significant bilateral bone loss over a short time period.

There are potential explanations other than disuse for the bone loss observed in this study, such as inflammatory osteolysis. Bone loss associated with inflammation has been demonstrated across a wide variety of acute and chronic infections, and other conditions including Charcot neuroarthropathy. This inflammatory osteolysis is thought to involve the pro-inflammatory mediators IL-1β, IL-6, and TNF-α^18,19^. Diabetes-related foot infections are associated with 105%, 47%, and 89% higher levels of IL-1β, IL-6, and TNF-α, respectively^20^, possibly contributing to the BMD losses observed in this study. Such inflammatory mediated osteolysis would be expected to result in a generalised loss of BMD. Although our study demonstrated BMD losses at all sites, only the losses at the total hip and femoral neck were significant. It is possible that, given the power limitation of this study, the combined effects of inflammatory osteolysis and reduced weight-bearing of the lower limbs resulted in significant BMD losses only being detected at these weight-bearing sites.

BMD losses reported here are significantly higher than described in longitudinal studies of BMD decline in normal aging^21–23^. The rate of bone loss in aging men is reported to be 0.5 to 1% per year, in early post-menopausal women up to 2% per year and late menopause 1% per year^21^. More specifically at the total hip, BMD declines by < 0.5% per year in pre-menopausal women and aging men and < 1% per year in post-menopausal women^22,23^. BMD losses described in our study are also more rapid than described in longitudinal studies in people with type 1 and type 2 diabetes, in which decreases in femoral neck BMD are less than 1% per year^24–28^. In fact, the rapid rate of BMD loss experienced at the femoral neck by participants in this study was comparable to that observed over longer durations in clinical settings such as the use of androgen deprivation therapy for prostate cancer which is associated with an established increased risk of osteoporosis and fractures^21^.

The BMD losses observed may have significant implications for the future development of osteoporosis and increased fracture risk in people with DFU. Given that 68% of participants had incompletely healed ulcers at 12 weeks requiring further offloading, continued losses in BMD may have occurred with longer follow up. Even if the high rates of BMD loss seen in this study were transient, the absolute losses of BMD sustained during the study period may still increase the risk of these complications in the long-term. Previous studies have shown that BMD losses associated with immobilisation of a lower limb following injury or surgery are either partially or completely irreversible^16,17^. This irreversibility is especially relevant to this study’s cohort who had a mean age of 62 years, as aging is associated with reduced ability to regenerate bone^29^. Therefore, it is possible that people hospitalised for DFU may not recover the associated acute BMD deficit. Unlike lower limb injuries which typically represent a discrete episode, DFU has a high recurrence rate, with 40% of people with a healed DFU experiencing another foot ulcer within a year^2^. If BMD losses were sustained following each episode of DFU, the cumulative reduction in BMD may be substantial and contribute to increased risk of fractures in the future. In elderly women, a 5% decline in BMD at the femoral neck has been shown to translate to a 40% increased risk of any fracture, independent of baseline BMD^30^. Given that both types of diabetes are associated with an established increased fracture risk particularly at the hip^5–7^, this finding of accelerated bone loss in people with DFU will likely potentiate this risk, further highlighting the clinical implications of this study. This fracture risk may be further exacerbated by additional risks for falling more common in people with DFU, such as peripheral neuropathy, visual impairment, gait disorders and poor mobility as a result of dressings, orthoses or footwear^5^. While there is minimal data regarding fracture risk specifically in people with diabetes and DFU, a retrospective study of US veterans with diabetes found a higher risk of falls and fractures in people with diabetes and DFU compared to those with diabetes alone^31^.

This study highlights a potential complication of DFU which has been previously unrecognised and may contribute to the already heavy burden of comorbidities experienced by people with this condition. Whilst further research is required, people hospitalised for DFUs may benefit from BMD monitoring and evaluation of falls risk. Should this accelerated bone loss be confirmed in larger prospective studies, further work is needed to identify effective therapeutic interventions, which may include nutritional supplementation, physiotherapy and/or anti-resorptive agents which have been used in the management of disuse osteoporosis associated with profound immobilisation^32^. In addition, Pakarinen et al. demonstrated that zoledronic acid prevented losses in total hip and femoral neck BMD in people required to immobilise their foot for acute Charcot neuroarthropathy^15^ suggesting the potential for similar therapies in DFU.

This study had some limitations. The small number of participants reduced the statistical power of this study. This may explain why small changes in fat and lean mass were not observed. A longer duration of follow-up may have been associated with greater changes in study measures including BMD, fat mass and lean mass. The lack of an appropriate control group means this study is unable to determine whether the BMD losses were independently associated with the hospitalisation and management of DFU. Lastly, the role of changes in bone metabolism and inflammation in the BMD losses observed were not explored. Future studies should address these limitations.

A significant decline in bilateral total hip and femoral neck BMD was observed in participants 12 weeks following hospitalisation for DFU. This novel finding potentially has important impacts on fracture risk for people with DFU, who experience a greater risk of falls and have an established increased risk of neck of femur fractures due to their diabetes. Future studies of longer duration and with an appropriate control population are required to investigate underlying mechanisms including whether these changes were due to the offloading and reduced weight-bearing associated with DFU management. Furthermore, studies are required to confirm the clinical implications of these changes and the efficacy of fracture prevention measures in people with DFU.
